# Age and Stroke Severity Matter Most for Clinical Outcome in Acute Arteriosclerotic Tandem Lesions

**DOI:** 10.3390/jcm13082315

**Published:** 2024-04-17

**Authors:** Cyrill Huber, Jatta Berberat, Renske Sassenburg, Stefanie Pflugi, Javier Anon, Michael Diepers, Lukas Andereggen, Timo Kahles, Andreas R. Luft, Krassen Nedeltchev, Luca Remonda, Philipp Gruber

**Affiliations:** 1 Department of Neuroradiology, Kantonsspital Aarau, University of Zurich, Tellstrasse 21, 5001 Aarau, Switzerland; cyrill.huber@ksa.ch; 2 Department of Neuroradiology, Kantonsspital Aarau, 5001 Aarau, Switzerland; jatta.berberat@ksa.ch (J.B.); renskesassenburg@gmail.com (R.S.); javier.anon@ksa.ch (J.A.); michael.diepers@ksa.ch (M.D.); luca.remonda@ksa.ch (L.R.); 3 Department of Neurosurgery, Kantonsspital Aarau, University of Bern, 3012 Bern, Switzerland; lukas.andereggen@ksa.ch; 4 Department of Neurology, Kantonsspital Aarau, University of Basel, 4001 Basel, Switzerland; timo.kahles@ksa.ch; 5 Department of Neurology, University Hospital Zurich, University of Zurich, 8952 Zurich, Switzerland; andreas.luft@usz.ch; 6 Cereneo, Center for Neurology and Rehabilitation, 6354 Vitznau, Switzerland; 7 Department of Neurology, Kantonsspital Aarau, University of Bern, 3012 Bern, Switzerland; krassen.nedeltchev@ksa.ch

**Keywords:** tandem lesion, acute ischemic stroke, epidemiology

## Abstract

**Background**: Tandem lesions (TLs) cause up to 15–30% of all acute ischemic strokes (AISs). Endovascular treatment (EVT) is regarded as the first-line treatment; however, uncertainties remain with respect to the treatment and predictive outcome parameters. Here, we aimed to identify the clinical and demographic factors associated with functional short- and long-term outcomes in AIS patients with arteriosclerotic TLs undergoing EVT. **Methods**: This was a retrospective, mono-centric cohort study of 116 consecutive AIS patients with arteriosclerotic TLs who were endovascularly treated at a stroke center, with analysis of the relevant demographic, procedural, and imaging data. **Results**: A total of 116 patients were included in this study, with a median age of 72 years (IQR 63–80), 31% of whom were female (n = 36). The median NIHSS on admission was 14 (IQR 7–19), with a median ASPECT score of 9 (IQR 8–10) and median NASCET score of 99% (IQR 88–100%). A total of 52% of the patients received intravenous thrombolysis. In 77% (n = 89) of the patients, an antegrade EVT approach was used, with a good recanalization (mTICI2b3) achieved in 83% of patients (n = 96). Symptomatic intracerebral hemorrhage occurred in 12.7% (n = 15) of patients. A favorable outcome (mRS0–2) and mortality at 3 months were obtained for 40% (n = 47) and 28% of patients (n = 32), respectively. Age and NIHSS on admission were strongly associated with outcome parameters. Diabetes mellitus and previous neurological disorders were independently associated with long-term mortality (median 11 months, IQR 0–42). **Conclusions**: Younger age, lower stroke severity, and good recanalization were found to be independently associated with a favorable outcome. In contrast, older age, higher stroke severity, previous neurological disorders, and diabetes were correlated with mortality. The endovascular treatment of acute arteriosclerotic tandem lesions is feasible and relatively safe.

## 1. Introduction

In 15% to 30% of patients with acute ischemic stroke (AIS), intracranial large-vessel occlusion (LVO) occurs with a concomitant extracranial carotid artery pathology [1,2]. If treated only with the best medical treatment, including intravenous thrombolysis (IVT), these tandem lesions (TLs) are associated with a poor functional outcome in up to 80% of affected patients [3,4]. Based on several retrospective reports and meta-analyses [1,4,5], endovascular treatment (EVT) is considered the first-line therapy in the acute management of TLs, with higher odds of a favorable outcome [6,7]. However, TLs are technically challenging lesions, and there are uncertainties regarding the best acute endovascular management and notable heterogeneity in the different approaches [2,8,9]. There is still a lack of clinical and procedural data supporting the treatment of these complex lesions.

Given this very complex pathology, it is of great importance to identify the clinical, procedural, and demographic factors that could impact the outcomes of acute TL patients in order to define clear decision-making and treatment concepts for clinical approaches.

Thus, we aimed to identify the clinical, procedural, and demographic factors associated with functional short- and long-term outcomes in a mono-center cohort study of 116 consecutive AIS patients with exclusively arteriosclerotic TLs, all of whom received EVT, for the improved selection and treatment of TL patients.

## 2. Material and Methods

### 2.1. Patients and Study Design

In this retrospective, mono-arm, mono-center cohort, we analyzed the epidemiological, clinical, procedural, and imaging data of all patients with AIS of the anterior circulation caused by tandem extracranial and intracranial steno-occlusive lesions, who received acute endovascular treatment at our comprehensive stroke center between January 2009 and December 2022. Patients < 18 years of age or with TLs due to dissection or involving posterior circulation were excluded from this analysis. The study was approved by the local ethics committee with informed consent waived, in accordance with article 34 of the federal act on research involving human beings.

### 2.2. Methods

Epidemiological data, including cardiovascular risk factors (arterial hypertension, dyslipidemia, atrial fibrillation, smoking, diabetes mellitus), and clinical data such as the National Institute of Health Stroke Scale (NIHSS) score, were retrospectively reviewed and collected from the medical reports of our hospital. Previous neurological disorder was defined as any neurological disease, including previous strokes. We reviewed computed tomography (CT) scans on admission and follow-up, digital subtraction angiography (DSA) and neurovascular ultrasound (US) reports for procedural and imaging data to assess the Alberta Stroke Program Early CT (ASPECT) score, the American Society of Intervention and Therapeutic Neuroradiology (ASITN) Collateral Score, and the degree of carotid stenosis according to the North American Symptomatic Carotid Endarterectomy Trial (NASCET). Final intracranial revascularization was assessed using the modified Thrombolysis in Cerebral Infarction (mTICI) scale. Intracranial hemorrhage (ICH) with a clinical worsening of the NIHSS by 4 points was defined as symptomatic ICH (sICH). Stent patency was assessed by ultrasound within 24 h following endovascular recanalization, and, subsequently, at the 3-month follow-up visit.

### 2.3. Outcome Measures

The primary outcome measures were a good functional outcome, defined as a modified Rankin Scale (mRS) score of 0–2, and mortality (mRS 6) from any cause at short-term follow-up (FU), defined as the first clinical FU after hospitalization, with a median of 3 months (IQR 0–4), or at long-term FU, defined as the last clinical FU, with a median of 11 months (IQR 0–42, minimum: 0 months, maximum: 149 months).

### 2.4. Statistical Analysis

The statistical analysis was conducted using Stata/IC 14.2 (StataCorp, College Station, TX, USA). Descriptive statistics were performed with discrete variables expressed as frequencies and non-normally distributed variables expressed as medians with interquartile ranges (IQR 25th–75th percentiles). Either the *t*-test or the Mann–Whitney test was used to compare the two groups. Univariate and multivariate logistic models with an odds ratio (OR) and 95% confidence intervals (95%) for a good outcome and mortality were performed. *p* < 0.05 was considered statistically significant.

## 3. Results

### 3.1. Demographic, Clinical, and Imaging Characteristics

The median age of the 116 patients was 72 years (IQR 63–80), and 31% (n = 36) were women. The median clinical FU time was 11 months (IQR 0–42) (Table 1). The most common cardiovascular risk factor was arterial hypertension (79%), followed by smoking (49%) and dyslipidemia (46%) (Table 1). The median NIHSS score on admission was 14 (7–19), with both hemispheres equally affected (52% for the right hemisphere and 48% for the left hemisphere) (Table 2). A total of 27% (n = 32) were “wake-up” strokes. The median NASCET grading score of the internal carotid artery (ICA) stenosis was 99% (IQR 90–100). The most common intracranial occlusion site was the M1 segment of the middle cerebral artery (MCA) (58%). In all patients, a CT scan was performed on admission. The median ASPECT score was nine (IQR 8–10), with a median ASITN collateral grading of three (IQR 2–3) (Table 2).

### 3.2. Procedural Characteristics, Success Rate, and Complications

All patients received an endovascular treatment, with 77% (n = 89) receiving an antegrade approach, in which the proximal lesion (bulbar) was treated first, and the distal lesion (intracranial) was treated afterwards. The other 23% (n = 27) of patients received a retrograde approach. The endovascular approach (antegrade or retrograde) was at the discretion of the treating interventionalist. The antegrade approach was more commonly performed in M2 segment occlusions (90% vs. 67%, *p* = 0.046) (Table 2). However, no other association was found with the occlusion side, clinical severity, technical considerations, or clinical outcome. Slightly more than half of all patients (52%; n = 60) received an IVT prior to treatment with a body-weight-adjusted tissue-type plasminogen activator (rtPA, Actilyse, Boehringer, Ingelheim am Rhein, Germany). In almost half of the patients (48%; n = 56), the procedure was carried out under general anesthesia (GA), which was associated with a higher NIHSS on admission (median NIHSS 16 (IQR 10–19), *p* = 0.01) and with carotid T-occlusion (64% vs. 44%, *p* = 0.07). No difference in procedure time, time-to-revascularization, or symptom-to-groin puncture was observed in patients who received GA compared to patients who did not. All the endovascular procedures were performed via femoral access. The median symptom-to-groin puncture time was 277 min (IQR 186–412 min), and the median symptom-to-revascularization time was 403 min (IQR 318–564 min), with a median procedure time of 137 min (IQR 96–175 min). In most of the patients, a carotid stent was placed in the bulbar ICA (94%; n = 111). The most used stent system was the Cristallo Ideale (Invatec Medtronic, Roncadelle, Italy), a self-expanding carotid stent system with a hybrid cell design (47%; n = 55), followed by the self-expanding closed-cell Carotid Wallstent (Boston Scientific, Marlborough, MA, USA) stent system (28%; n = 32), and, finally, the CASPER (Microvention, Aliso Viejo, CA, USA) double-layer stent system (20%; n = 23). Peri-interventionally, slightly more than half of the cases received a renal and weight-adapted IV bolus with heparin (58%; n = 67) and an IV bolus with aspirin (500 mg) (55%; n = 64). For a first attempt, the intracranial lesions were most commonly treated with mechanical thrombectomy (61%; n = 71), followed by the aspiration technique only (20%; n = 23). Intra-arterial (IA) thrombolysis was only performed in four cases (4%). In 18 cases (16%), no additional intracranial intervention was performed since the intracranial lesion had either resolved during the intervention (9%; n = 8) or the occlusion site was ultimately too distant (9%; n = 10). For MCA M2 segmental occlusions, intracranial procedures in general (30% vs. 1.2%, *p* < 0.0001) and mechanical thrombectomy specifically (43.3 vs. 67.4%, *p* = 0.02) were less frequently performed compared to other intracranial occlusions. Otherwise, no significant differences were observed between the revascularization techniques and the anatomical occlusion sites. Good recanalization (mTICI 2b-3) was achieved in 83% (n = 96) of the patients. The stent patency rate was 93% (n = 97) at first FU, and there was no difference between the various stent designs used. The median residual stenosis rate was low (NASCET 0% (IQR 0–0%)). Early in-stent thrombosis occurred in only six (5%) patients (in five within 24 h and in one patient after 5 days). Intracranial hemorrhage (ICH) occurred in 29% (n = 33) of the patients and reflected symptomatic ICH in 12.9% (n = 15) of the patients. ICH occurred significantly more often in cancer patients than in non-cancer patients (56% vs. 24%, *p* = 0.0063).

Interestingly, the use of IV or IA thrombolysis and the use of antiplatelet or anticoagulant agents were not associated with sICH.

In six patients, emergency decompressive craniectomies were performed due to malignant media infarction or ICH. Most of these patients were younger (mean age 62 years (IQR 60–66 years)).

**Figure 1:**
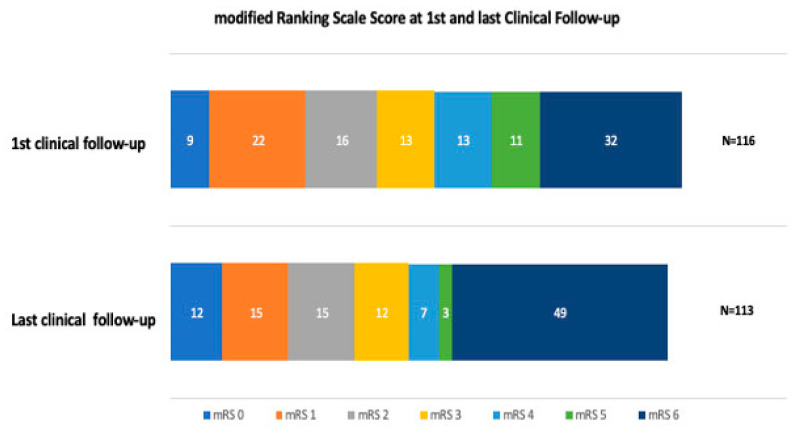
Within the first FU and last clinical FU, the proportion of patients who had a favorable outcome (mRS 0–2) decreased from 49 patients to 42 patients, respectively. Within the first and the last clinical FU, the proportion of deceased patients increased, and within the last FU, a large number of patients from the cohort died. Among those patients, 21 were older than 79 years of age; thus, 70% of the group of > 79-year-old patients were deceased. Half of the patients died during their hospital stay, and only a few died within the first FU period.

The median duration of hospital stay was 10 days (IQR 6–16 days), with a median discharge NIHSS of six (IQR 2–13) and an in-hospital mortality rate of 20% (n = 23) (Table 3). The most common reasons for in-hospital mortality were high stroke severity on admission, ICH, and pneumonia. At the first clinical follow-up, the mean mRS score was three (IQR 1–6), and mortality increased from 19.8% at discharge to 27.6% at 3 months FU (Figure 1). A favorable outcome (mRS 0–2) was achieved for 41% (n = 47) of the patients at 3 months. Of these, 12 (10%) patients had an excellent outcome (mRS0). At long-term FU, the median mRS increased to four (IQR 2–6). A relatively high all-cause mortality rate was observed in this cohort, especially within the long-term follow-up, by which time 43% (n = 49) of the patients had died (Figure 1). However, half died in hospital, and only a few died within the first FU period (Table 3). There was a very low rate (less than 1%) of re-ischemic event in the same territory within the last follow-up period, as well as a very low rate (less than 1%) of retreatment within the last follow-up period. Within FU, 17 (15%) of the patients developed seizures (Table 3).

There was an association between the clinical and imaging features in terms of functional outcome and mortality at short-term and long-term follow-up.

In patients with a favorable outcome (mRS 0–2), diabetes mellitus (DM) was less frequent (*p* = 0.017), there were fewer previous neurological disorders (*p* = 0.017), ICH (*p* = 0.012) and sICH (*p* = 0.001) were less common, a faster procedure time was achieved (136 min, IQR 88–163 min; *p* = 0.03), and GA was less frequently (*p* = 0.0034) used. For all other demographic, imaging, and procedural variables, there were no differences found between patients with and without favorable outcomes. Interestingly, no difference was found either for prior IVT nor for the EVT approach (antegrade vs. retrograde). However, the proportion of patients with a favorable outcome (mRS 0–2) decreased during the long-term FU period (Figure 1).

In our multivariate logistic regression analysis, younger age, lower NIHSS on admission, and shorter procedural time were independently associated with a favorable outcome.

Patients who died prior to the first clinical FU at 3 months were older (80 years (IQR 74–84), *p* = 0.0002), presented with a higher NIHSS on admission (18 (IQR 14–20), *p* = 0.0001), and suffered more commonly from DM (35% vs. 17%, *p* = 0.039), nephropathies (35% vs. 10%, *p* = 0.0013), chronic lung disease (25% vs. 8%, *p* = 0.0173), obesity (38% vs. 19%, *p* = 0.038), and previous neurological disorders (38% vs. 8%, *p* = 0.0002). As a consequence, sICH was more common in this group of patients (31% vs. 6%, *p* = 0.0008%). However, there were no differences regarding imaging data, EVT technique, or prior IVT.

In our multivariate logistic regression analysis, older age, higher NIHSS on admission, and previous neurological disorders were independently associated with mortality within the first clinical FU period.

Among patients who died within the last clinical FU period, DM (35% vs. 17%, *p* = 0.039), atrial fibrillation (35% vs. 13%, *p* = 0.005), coronary heart disease (20% vs. 4%, *p* = 0.046), peripheral artery disease (PAD, 18% vs. 6%, *p* = 0.046) and nephropathies (35% vs. 10%, *p* = 0.0013), and previous neurological disorders (31% vs. 6%, *p* = 0.0006) were significantly more common compared to the surviving patients. Among those patients, 21 were older than 79 years of age, meaning that 70% of the group of > 79-year-old patients died. This group of patients was also significantly older (78 years (IQR 73–83), *p* ≤ 0.0001) and presented with a higher NIHSS score on admission (18 (IQR 12–20), *p* = 0.0001) compared to the surviving patients. There were no differences regarding the EVT technique or prior IVT. In the logistic multivariate analysis, older age, higher NIHSS on admission, DM, and previous neurological disorders were independently associated with mortality within the last FU period (Table 4). The same was true for very old patients (>79 years) and patients with a high NIHSS score on admission (NIHSS score > 16).

## 4. Discussion

In this retrospective cohort study of 116 AIS patients with arteriosclerotic TLs, age and stroke severity (NIHSS score on admission) were independently associated with a favorable outcome, as well as short- and long-term mortality. Previous neurologic disorders, older age, higher NIHSS on admission, and DM were strongly associated with long-term mortality. Except for shorter procedural time, which was associated with a favorable outcome, no associations with a favorable outcome or mortality were found for differing procedural and imaging features. Overall, the cohort had a relatively high long-term mortality rate despite good initial ASPECT scores, a high recanalization rate, and low numbers of passes.

Our cohort was in the upper range of reported median ages compared to other TL studies [10,11,12,13,14]. However, gender, NIHSS on admission, procedural time, recanalization rate, and sICH and ICH rates were similar to those found in other reports [1,4,10,15,16]. In this cohort, a low recurrent stroke rate of 1% was found, which was lower than the recurrent stroke rate of a recent large register study of 6% [1].

Our cohort had an overall high successful recanalization rate (TICI 2b3) of 83% and a favorable clinical outcome rate (mRS 0–2) of 41%, supporting the findings of previous cohort studies, such as the pooled individual data of the TITAN registry [11], that suggested that EVT with ICA stenting in TLs is feasible and safe [8,10,12,15,16,17,18,19]. In addition, our results were comparable to the outcome data for common AIS patients with LVO, as reported by the HERMES trial, of 46% [7]. Contrary to other reports, where a retrograde approach (first intracranial thrombectomy followed by an extracranial ICA stenting) was associated with better outcomes [1,9], we did not find any difference regarding the procedural data, bleeding rate, or outcome parameters based on the use of a retrograde or antegrade approach.

In our cohort, there was no difference between patients with prior IVT use and patients without prior IVT use in terms of favorable outcomes, ICH, sICH, or any other technical features, which was similar to previous observations reported by the TITAN study group [4], indicating that prior IVT use is safe. However, prior IVT might be of limited effect due to the extensive clot burden in TL patients [3,20].

Since nearly all of our patients received extracranial stenting, there was a high stent-patency rate of over 90%. We observed only a few early in-stent thromboses and a few retreatments in the longer-term FU. There was a low sICH rate, and only one patient with an ischemic re-event in the same territory. Despite a relatively high rate of ICH, in line with other reports [10,15], these findings indicated that stent placement in an acute setting is feasible and safe, without a high rate of restenosis or sICH. In our cohort, contralateral stenosis was not a predictive marker for any outcome parameter, as postulated by others [21].

Our cohort had a relatively high long-term mortality, and nearly half of the patients died within the last FU period. A large proportion of the deceased patients died immediately, during their hospital stay (46%), while a smaller proportion died within the first clinical FU period (18%). In the long run, many of the remaining patients of this group died (36%). Factors that had an influence on the mortality rate included older age, higher NIHSS score, DM, and previous neurological disorders. Interestingly, demographic and clinical features had a significant impact for both the mortality rate within the first clinical FU period and within the long-term clinical FU period. However, procedural and imaging features only showed a limited impact on favorable outcome rates within the 3-month FU period. These findings indicated that, for a longer time horizon post-stroke survival, age, comorbidities, and physical fitness are of the utmost importance [22], and periprocedural features are less so. Further, as currently reported for common LVO AIS patients [23], very old TL patients (>79 years) were much more likely to die within the follow-up period, which raises the question of how aggressively an EVT treatment should be utilized in this group of patients [24].

Furthermore, DM was revealed to be a strong independent parameter of long-term mortality. Patients with DM have an increased risk of poor outcomes following a stroke [25,26,27].

In conclusion, our results indicated that younger TL patients and TL patients with fewer neurological deficits are more likely to benefit from an acute EVT. In addition, a good recanalization rate increases the likelihood of a favorable outcome. Thus, our data may help to optimize EVT selection criteria in TL patients and inform how aggressive an EVT treatment should be, which could be important when resources are limited. Further, our data suggested that demographic and clinical factors play an important role in the outcome and the selection of TL patients for EVT. However, more prospective data are needed to support these findings.

The limitations of this study include its retrospective, observational design and lack of a standardized procedural protocol. Thus, there are limitations due to missing data, unbalanced variables, and the observation time of patients not being pre-specified and ranging from a few months to several years.

## 5. Conclusions

Our data showed that younger age and lower stroke severity, as well as good recanalization, are independently associated with favorable outcomes. In contrast, older age, higher stroke severity, previous neurological disorders, and diabetes are correlated with mortality. The endovascular treatment of acute arteriosclerotic tandem lesions is feasible and relatively safe.

## Figures and Tables

**Table 1:** Summary of the demographic characteristics of the patients.

	All Patients (n = 116)
Women, no. (%)	36 (31%)
Age in years, median (IQR)	72 (63–80)
Medical History	
Hypertension, no. (%)	92 (79%)
Arterial fibrillation, no. (%)	27 (23%)
Coronary heart disease, no. (%)	14 (12%)
PAD, no. (%)	14 (12%)
Dyslipidemia, no. (%)	53 (46%)
Diabetes mellitus, no. (%)	25 (22%)
Obesity, no. (%)	28 (24%)
Nephropathy, no. (%)	19 (16%)
Lung disease, no. (%)	15 (13%)
Sleep apnea syndrome, no. (%)	11 (10%)
Cancer, no. (%)	18 (16%)
Previous neurological disease, no. (%)	19 (17%)
Smoking, no. (%)	57 (42%)
Alcohol abuse, no. (%)	10 (9%)
Previous Medication	
Previous antiplatelet therapy, no. (%)	32 (28%)
Previous oral anticoagulation, no. (%)	0 (0%)
Previous NOAC, no. (%)	6 (5%)

IQR, interquartile range; no., number; NOAC, non-vitamin K oral anticoagulants; PAD, peripheral artery disease.

**Table 2:** Summary of the clinical, imaging, and procedural data.

	n = 116
Clinical data	
Left side, no. (%)	55 (47%)
Wake-up constellation, no. (%)	32 (28%)
NIHSS on admission, median (IQR)	14 (7–19)
Imaging data	
ASPECT score, median (IQR)	9 (8–10)
ASITN score, median (IQR)	2 (2–3)
Carotid T occlusion, no. (%)	25 (22%)
MCA M1 occlusion, no. (%)	67 (58%)
MCA M2 occlusion, no. (%)	30 (26%)
ACA A1 occlusion, no. (%)	8 (9%)
ACA A2 occlusion, no. (%)	5 (4%)
NASCET score in percent, median (IQR)	99 (88–100)
Contralateral ICA stenosis (>60%), no. (%)	15 (13%)
Procedural data	
Prior IV rtPA thrombolysis, no. (%)	60 (52%)
General anesthesia, no. (%)	56 (48%)
Peri-interventional aspirin bolus, no. (%)	64 (55%)
Peri-interventional heparin bolus, no. (%)	67 (58%)
Antegrade, no. (%)	89 (77%)
Number of passes, no. (%)	1 (1–2)
Successful recanalization (mTICI Score 2b–3), no. (%)	96 (83%)
Symptom-to-groin time, median (IQR)	277 (186–412)
Procedural time, median (IQR)	137 (96–175)
Symptom-to-revascularization time, median (IQR)	403 (318–564)
Thrombectomy, no. (%)	71 (61%)
Aspiration, no. (%)	23 (20%)
IA-Thrombolysis, no. (%)	4 (4%)
No intervention, no. (%)	18 (16%)
Cristallo ideal stent, no. (%)	55 (47%)
Carotid wallstent, no. (%)	32 (28%)
CASPER stent, no. (%)	23 (20%)
Decompressive hemicraniectomy	6 (5%)

ACA, anterior cerebral artery; ASITN, American Society of Intervention and Therapeutic Neuroradiology; ASPECT, Alberta Stroke Program Early CT; ia, intra-arterial; IQR, interquartile range; IV, intravenous; MCA, middle cerebral artery; mTICI score; modified Thrombolysis in Cerebral Infarction; NIHSS score, National Institute of Health Stroke Scale; no., number.

**Table 3:** Summary of the outcome and follow-up parameters.

	n = 116
FU in months, median (IQR)	11 (0–42)
1st clinical FU in months, median (IQR)	3 (0–4)
mRS at 1st FU, median (IQR)	3 (1–6)
mRS at last FU, median (IQR) ^†^	4 (2–6)
Favorable outcome at 1st FU, no. (%)	47 (40.5%)
Favorable outcome at last FU, no. (%)	42 (37.2%)
Mortality rate within 1st FU, no. (%)	32 (27.6%)
Mortality rate last FU, no. (%) ^†^	49 (43.4%)
In-hospital death, no. (%)	23 (19.8%)
sICH, no. (%)	15 (12.9%)
ICH, no. (%)	33 (28.7%)
Stent patency, no. (%)	97 (93.3%) **
Re-PTA within last FU, no. (%)	1 (0.8%)
Any re-ischemic event, no. (%)	6 (5.2%)
Re-ischemic event in the same territory, no. (%)	1 (0.8%)
Newly occurring seizures, no. (%)	17 (14.7%)

FU, follow-up; mRS, modified Ranking Scale; ** n = 103; PTA, percutaneous transluminal angioplasty; ^†^ n = 113; ICH, intracranial hemorrhage; sICH, symptomatic hemorrhage.

**Table 4:** Logistic multivariate analysis of outcome parameters.

Parameters *	Favorable Outcome OR (CI 95%)	Mortality 1st FU OR (CI 95%)	Mortality Last FU OR (CI 95%)
Age	0.9 (0.9–0.9), *p* = 0.009	1.1 (1.0–1.2), *p* = 0.008	1.1 (1.1–1.2), *p* = 0.000
NIHSS on admission	0.9 (0.9–9), *p* = 0.046	1.2 (1.1–1.3), *p* = 0.001	10.6 (3.1–35.8), *p* = 0.000
Diabetes mellitus	0.3 (0.1–1.0), *p* = 0.059	2.0 (0.6–7.0), *p* = 0.275	8.6 (2.0–36.5), *p* = 0.003
Pre. neurol. disorders	0.4 (0.1–1.8), *p* = 0.253	6.5 (1.7–25.5), *p* = 0.007	8.2 (1.8–37.9), *p* = 0.007
CDH	0.2 (0.0–1.97), *p* = 0.173	-	1.9 (0.3–14.8). *p* = 0.501
Atrial fibrillation	-	-	3.6 (0.8–15.0), *p* = 0.081
PAD	-	-	1.9 (0.2–14.9), *p* = 0.562
Nephropathy	-	1.6 (0.38–6.79), *p* = 0.65	0.4 (0.1–2.3), *p* = 0.318
Lung disease	-	2.1 (0.49–8.69), *p* = 0.321	-
Adiposity	-	2.8 (0.78- 10.12), *p* = 0.11	-
Procedural time	0.9 (0.9–1.0), *p* = 0.001	-	-

CI 95%, 95% confidence interval; OR, odds ratio; CHD, coronary heart disease; PAD, peripheral artery disease; prev. neurol. disorders, previous neurological disorders; * in the univariate analysis, all parameters were significant.

## Data Availability

The data presented in this study are available on request from the corresponding author.
